# Continuous renal replacement therapy for safe and adequate voriconazole intravenous treatment: enough reason to be confident?

**DOI:** 10.1186/s13054-015-0946-1

**Published:** 2015-05-27

**Authors:** Patrick M Honore, Rita Jacobs, Inne Hendrickx, Elisabeth De Waele, Viola Van Gorp, Herbert D Spapen

**Affiliations:** ICU Department, Universitair Ziekenhuis Brussel – Vrije Universiteit Brussels, 101 Laarbeeklaan, 1090 Jette, Brussels Belgium

Voriconazole is a first-line agent for treatment of systemic mycotic infections. However, intravenous use is contraindicated in patients with creatinine clearance <50 ml/minute because of accumulation of the toxic vehicle sulfobutylether-beta-cyclodextrin sodium [1, 2]. In a recent issue of *Critical Care*, Kiser and colleagues furnished convincing pharmacological evidence that sulfobutylether-beta-cyclodextrin sodium but not voriconazole was effectively removed by continuous veno-venous hemofiltration (CVVH). They concluded that standard intravenous voriconazole doses could be safely used when patients were placed under continuous renal replacement therapy [3].

We acknowledge the clinical relevance of this study but advocate a more balanced appraisal of the results. First, Kiser and colleagues applied CVVH doses ranging from approximately 25 to 75 ml/kg/hour in a small group of patients. This approach might introduce significant difference in substance elimination and does not conform to routinely used CVVH doses. Second, the study showed that sulfobutylether-beta-cyclodextrin sodium, being a middle molecular weight substance, was highly and dose-dependently eliminated by convection. However, diffusion-based continuous renal replacement therapy arguably will produce equally effective elimination. Third, if CVVH is performed without high flux or high cutoff membranes, convective capacity may rapidly falter due to a decrease in membrane porosity. This can be avoided by using regional citrate anticoagulation, which was not applied in this study.

Therefore, we recommend using CVVH (to privilege convective drug elimination) at a dose of 35 ml/kg/hour (to assure a minimal delivered dose of 25 ml/kg/hour) under regional citrate anticoagulation (to consolidate filter function) for permitting safe and adequate intravenous voriconazole treatment.

## Abbreviations

CVVH: continuous veno-venous hemofiltration
